# Intracerebral Hemorrhage in Aging: Pathophysiology, Clinical Challenges, and Future Directions

**DOI:** 10.3390/life15101569

**Published:** 2025-10-08

**Authors:** Esra Zhubi, Andrea Lehoczki, Peter Toth, Dominika Lendvai-Emmert, Levente Szalardy, Bence Gunda

**Affiliations:** 1 Department of Neurology, Semmelweis University, 1083 Budapest, Hungary; esra.zhubi@phd.semmelweis.hu; 2 Institute of Preventive Medicine and Public Health, Semmelweis University, 1089 Budapest, Hungary; 3 Fodor Center for Prevention and Healthy Aging, Semmelweis University, 1085 Budapest, Hungary; 4 Department of Neurosurgery, Medical School, University of Pecs, 7623 Pecs, Hungary; 5 Department of Neurosurgery, University of Oklahoma Health Sciences Center, Oklahoma City, OK 73104, USA; 6 Department of Neurology, Albert Szent-Györgyi Medical School, University of Szeged, 6725 Szeged, Hungary; szalardy.levente@med.u-szeged.hu

**Keywords:** aging, cerebral amyloid angiopathy, cerebral microbleeds, intracerebral hemorrhage, small vessel disease, vascular senescence

## Abstract

Spontaneous intracerebral hemorrhage (ICH) is a devastating form of stroke, disproportionately affecting older adults and is associated with high rates of mortality, functional dependence, and long-term cognitive decline. Aging profoundly alters the structure and function of the cerebral vasculature, predisposing the brain to both covert hemorrhage and the development of cerebral microbleeds (CMBs), small, often subclinical lesions that share common pathophysiological mechanisms with ICH. These mechanisms include endothelial dysfunction, impaired cerebral autoregulation, blood–brain barrier breakdown, vascular senescence, and chronic inflammation. Systemic factors such as age-related insulin-like growth factor 1 (IGF-1) deficiency further exacerbate microvascular vulnerability. CMBs and ICH represent distinct yet interconnected manifestations along a continuum of hemorrhagic small vessel disease, with growing recognition of their contribution to vascular cognitive impairment and dementia (VCID). Despite their increasing burden, older adults remain underrepresented in clinical trials, and few therapeutic approaches specifically target aging-related mechanisms. This review synthesizes current knowledge on the cellular, molecular, and systemic drivers of ICH and CMBs in aging, highlights diagnostic and therapeutic challenges, and outlines opportunities for age-sensitive prevention and individualized care strategies.

## 1. Introduction

Spontaneous intracerebral hemorrhage (ICH) is a devastating form of stroke, resulting from bleeding into the brain parenchyma [1,2]. Although it accounts for only 10–15% of all strokes, ICH carries the highest mortality and morbidity among all stroke subtypes [3,4]. Survivors often face profound and persistent functional dependence, long-term cognitive impairment, and diminished quality of life. As global populations continue to age, the burden of ICH is projected to rise sharply. Individuals over the age of 75 now represent a substantial and growing proportion of ICH cases, making it imperative to understand how aging itself [5], beyond traditional vascular risk factors such as hypertension or anticoagulant use, predisposes the brain to hemorrhagic injury [6].

ICH is increasingly recognized not as a random catastrophic event, but as the endpoint of chronic vascular pathology, particularly cerebral small vessel disease (cSVD) [7,8,9,10], which is highly prevalent in older adults [11,12]. Mechanisms such as vascular endothelial dysfunction, impaired autoregulation, blood–brain barrier (BBB) disruption, and cellular senescence converge to compromise the structural integrity of the cerebral microvasculature. These changes lead to a state of progressive microvascular fragility, which not only predisposes to large parenchymal bleeds but also gives rise to cerebral microbleeds (CMBs), tiny foci of blood leakage that accumulate silently over time [13,14,15].

CMBs, detectable by susceptibility-weighted imaging (SWI) on magnetic resonance imaging (MRI) [16], are increasingly viewed as an integral part of the hemorrhagic spectrum of cSVD [13,14,15,16,17]. Although often labeled “silent,” CMBs are far from clinically irrelevant. Their presence correlates with increased risk of future symptomatic ICH and is strongly associated with vascular cognitive impairment and dementia (VCID) [18,19,20,21,22,23]. In many cases, CMBs coexist with or precede overt hemorrhagic stroke, and they share common risk factors and underlying mechanisms, including chronic hypertension, cerebral amyloid angiopathy (CAA) [24,25], and age-related endothelial dysfunction. As such, CMBs and ICH should not be regarded as distinct entities, but rather as different manifestations along a continuum of hemorrhagic vascular brain injury [26].

This convergence has critical implications for public health. While neurodegenerative diseases like Alzheimer’s disease (AD) remain difficult to prevent or modify at the population level, vascular contributors to cognitive decline, including CMBs and ICH, are, at least in theory, modifiable and even preventable [27]. Recognizing and targeting the early microvascular changes that preceded over hemorrhage offers a compelling opportunity for intervention, particularly in aging populations at highest risk [28].

This review synthesizes current knowledge on the mechanisms by which aging promotes cerebral hemorrhage, with a special focus on cellular and molecular drivers of microvascular weakening. We explore how cSVD underlies both CMBs and larger ICH, review age-specific clinical presentations and outcomes, and examine evolving diagnostic and therapeutic strategies. By bridging basic vascular biology with clinical gerontology and stroke medicine, we aim to highlight the critical need for age-sensitive, mechanism-informed approaches to reduce the burden of hemorrhagic stroke and related cognitive decline in older adults.

## 2. Pathophysiological Mechanisms Linking Aging to CMBs and ICH

**Figure 1:**
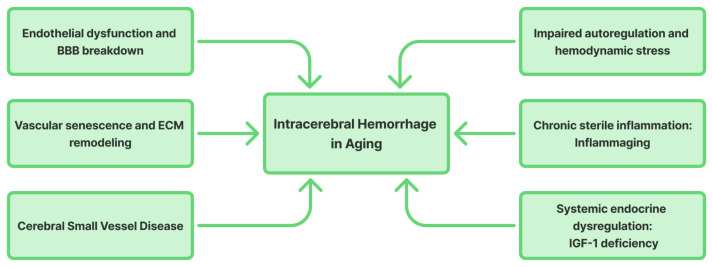
Pathophysiological mechanisms of intracerebral hemorrhage in aging brain (BBB: blood–brain barrier; ECM: extracellular matrix; IGF-1: insulin-like growth factor 1).

The vulnerability of the aging brain to both CMBs and spontaneous primary ICH [24] arise from a multifactorial process involving both local and systemic drivers of cerebrovascular aging [13,29,30,31,32,33,34,35,36,37,38,39,40]. These processes, many of which reflect hallmarks of aging, compromise the structural and functional integrity of cerebral vessels and contribute to the continuum of hemorrhagic cSVD [41]. The key pathophysiological mechanisms underlying these processes are illustrated in Figure 1.

### 2.1. Endothelial Dysfunction and Blood–Brain Barrier (BBB) Breakdown

With advancing age, endothelial cells in the cerebral microvasculature exhibit reduced nitric oxide (NO) bioavailability [42,43,44,45,46,47,48], impaired angiogenic signaling [49,50], and increased oxidative stress [51,52,53,54,55]. These changes impair endothelium-dependent vasodilation and compromise the integrity of the BBB [44,56,57,58,59,60,61,62,63,64], promoting leakage of plasma proteins and erythrocytes into parenchyma and inducing an inflammatory microenvironment [65,66]. BBB disruption is a key antecedent of both CMBs and ICH, particularly in the setting of fluctuating blood pressure [23].

### 2.2. Vascular Senescence and Extracellular Matrix (ECM) Remodeling

Cellular senescence [67,68], an important hallmark of aging [69,70,71,72,73,74,75,76,77], affects various components of the neurovascular unit [42,57,60,77,78,79,80], such as endothelial cells [81,82], pericytes, microglia [83] and astrocytes [84,85], and leads to the release of senescence-associated secretory phenotype (SASP) factors [86,87], including pro-inflammatory cytokines and matrix metalloproteinases (MMPs) [88]. These mediators degrade the extracellular matrix (ECM), weaken the basement membrane, and promote loss of vascular elasticity and cohesion [89,90]. The net effect is increased susceptibility to microvascular rupture. Increased presence of senescent cells has also been linked to endothelial dysfunction [42], BBB disruption [57,60,77,91,92,93] and genesis of CMBs [35].

### 2.3. CSVD: A Common Pathological Substrate

CMBs and ICH frequently arise from two major forms of cSVD [34,94,95]. Hypertensive arteriopathy, characterized by arteriolosclerosis, lipohyalinosis, fibrinoid necrosis, and microaneurysm formation, predominantly and typically affects deep perforating arteries and is a major driver of deep ICH and deep CMBs, such as those located in the thalamus, basal ganglia, and brainstem (particularly the pons); however, lobar hemorrhages (including ICH and CMBs) can also develop secondary to hypertensive arteriopathy [23,96,97,98,99,100,101]. On the other hand, cerebral amyloid angiopathy (CAA) [9,102,103,104] involves the deposition of β-amyloid predominantly in cortical and leptomeningeal vessels, leading to vessel wall weakening and consequent hemorrhages in the parenchyma (i.e., lobar CMBs and lobar ICH) as well as the leptomeninges (i.e., convexity subarachnoid hemorrhage (cSAH) and its chronic manifestation, cortical superficial siderosis (cSS) [105]. The presence of the above hemorrhagic alterations in a strictly lobar/superficial pattern (i.e., without deep hemorrhages) allows the clinical-radiological diagnosis of *probable CAA* as per the consecutive versions of the Boston criteria (most recently the v2.0), with the mixed (i.e., lobar and deep) hemorrhagic pattern being considered to be more likely due to hypertensive arteriopathy only, and the cerebellar hemorrhagic alterations being not counted [106]. CAA is strongly age-dependent and is closely linked to cognitive decline. In both pathologies (i.e., hypertensive arteriopathy and sporadic CAA, microvascular rupture is the culmination of chronic degenerative processes, compounded by systemic factors such as hypertension and impaired cerebral autoregulation. Indeed, the etiology of hemorrhage formation in CAA is likely multifactorial, complex, and expands far beyond pure fragility due to β-amyloid deposition. This is supported by the observations that only a portion of patients with histopathologically defined moderate-to-severe CAA develop hemorrhagic alterations [103], and that an increased risk for ICH development in adult patients with Down syndrome (with the trisomy of chromosome 21 resulting in an excess copy of the *amyloid precursor protein (APP)* and subsequent severe CAA) is not established [107].

Reports on hyperacute ICH captured serendipitously during imaging support the hypothesis that hematoma expansion both in hypertensive and CAA related ICH results from multiple sources of bleeding due to a cascade of secondary vessel ruptures with eccentric expansion rather than a single source and continuous bleeding with concentric expansion reflecting the global fragility of the cerebral vasculature [108,109,110].

### 2.4. Impaired Cerebral Autoregulation and Hemodynamic Stress

Cerebral autoregulation is the intrinsic ability of the brain to maintain stable cerebral blood flow (CBF) despite fluctuations in systemic blood pressure [111,112,113,114]. This protective mechanism operates across a wide range of mean arterial pressures (typically 60–150 mmHg) and is essential for shielding the fragile microcirculation from ischemic or hemorrhagic injury. Autoregulation is accomplished through tightly orchestrated myogenic, metabolic, and neurogenic mechanisms that dynamically adjust cerebrovascular tone. In aging, cerebral autoregulation becomes progressively compromised. This decline not only reduces the brain’s ability to maintain perfusion during hypotension but also increases its vulnerability to hypertensive injury, contributing directly to the pathogenesis of both CMBs and ICH [23,27]. Both older individuals and aged laboratory animals exhibit blunted myogenic responses of cerebral resistance vessels, limiting the ability of the vasculature to constrict or dilate in response to fluctuations in perfusion pressure [115,116]. This diminished responsiveness compromises the brain’s capacity to buffer hemodynamic stress, increasing vulnerability to both hypoperfusion and hyperperfusion, while age-related changes in hypertensive conditions further disrupt the physiological adaptive remodeling processes of cerebral blood vessels. Research indicates that older mammals exhibit compromised remodeling capacity, characterized by impaired prostanoid signaling pathways, dysfunctional transient receptor potential (TRP) channel activity, and defective calcium signaling mechanisms, all of which collectively impair vascular plasticity and adaptive responses.

#### 2.4.1. Myogenic Dysfunction: Loss of Pressure-Induced Vasoconstriction

The myogenic response, mediated by vascular smooth muscle cells (VSMCs), is a central pillar of cerebral autoregulation [117]. In response to increased intraluminal pressure, VSMCs contract to maintain constant flow and prevent overperfusion [118]. Aging impairs this response through multiple mechanisms. First, it was shown earlier that aging impairs hypertension-induced increased pressure-dependent Ca^2+^ signaling in cerebrovascular smooth muscle cells in a 20-HETE/TRPC channel-dependent manner, leading to decreased pressure-induced constriction of the vessels [119,120]. Age-related decline in insulin-like growth factor 1 (IGF-1) signaling may play a pivotal role in this dysfunctional response to increased intraluminal pressure [121]. Second, it is believed that VSMCs senescence and phenotypic switching reduce contractile capacity. Third, ECM stiffening and collagen accumulation diminish vessel compliance. As a result, cerebral arterioles in the aged brain become less capable of constricting in response to systemic pressure increases [122]. This myogenic failure allows excessive pressure to be transmitted downstream to capillaries and venules, elevating the risk of mechanical rupture and hemorrhage [117,123].

#### 2.4.2. Impaired Adaptation to Chronic Hypertension

In younger individuals, chronic hypertension induces adaptive remodeling of cerebral arterioles, including wall thickening, increased smooth muscle content, and upward resetting of the autoregulatory curve [124]. This is completed by the increased 20-HETE-dependent pressure-induced constriction of cerebral vessels mentioned above [120,125]. These changes expand the upper limit of autoregulatory protection, allowing the brain to tolerate higher systemic pressures without hyperperfusion injury [126,127,128]. However, in aging, this adaptive capacity is blunted [120,125]. Vascular remodeling becomes maladaptive, characterized by stiffening, fibrosis, and inward hypertrophic remodeling without preserved function [129,130]. The failure to appropriately reset the autoregulatory range in response to sustained hypertension leaves aged microvessels dangerously exposed during hypertensive surges [120,125]. This impaired adaptation explains why older adults with moderate or even well-controlled hypertension can still suffer from hemorrhagic lesions. In line with the notion that deep perforating arterioles are the most affected by chronic hypertensive arteriopathy and consequent impaired adaptation, hypertensive surge at presentation (defined as ≥180 mmHg systolic blood pressure (SBP) was found to be an independent predictor of deep ICH location [105]. Superficial cortical/leptomeningeal vessels are more prone to be burdened by CAA (especially in older people, thus preferentially leading to lobar CMBs, cSAH/cSS, and lobar ICH. However, the prevalence of chronic hypertension was invariably high in patients with both CAA-related and non-CAA-related ICH, suggesting it to be a common underlying risk factor for ICH development in both CAA and hypertensive arteriopathy [105].

#### 2.4.3. Functional Consequences: CMBs and ICH as Spectrum Manifestations

When cerebral autoregulatory mechanisms fail, cerebral perfusion becomes passively dependent on systemic blood pressure. In this vulnerable state, hypertensive episodes, especially nocturnal or paroxysmal surges, can exert abrupt and excessive mechanical stress on the cerebral microvasculature [117,131,132]. Fragile vessels, already compromised by cSVD, are unable to buffer these fluctuations, leading to microvascular injury [131]. This failure of autoregulatory protection is particularly consequential in deep brain regions affected by hypertensive arteriopathy, where sudden pressure increases can trigger focal arteriolar rupture and result in ICH. At the same time, repeated subclinical stress, especially in cortical regions burdened by CAA, promotes chronic leakage, hemosiderin deposition, and the progressive formation of CMBs [133,134]. Even moderate, transient fluctuations in blood pressure, in the absence of sustained hypertension, can produce cumulative vascular damage over time in older adults with impaired autoregulation.

The underlying pathophysiology involves a combination of myogenic dysfunction, which blunts pressure-induced vasoconstriction, and impaired vascular adaptation to chronic hypertension, which fails to shift the autoregulatory range upward [117]. Together, these mechanisms create a permissive environment for both chronic microvascular leakage and acute rupture. CMBs and ICH should thus be viewed as distinct yet interconnected manifestations along a shared continuum of hemorrhagic small vessel pathology, where the severity and distribution of damage are shaped by vessel size, the presence of arteriolosclerosis or CAA, and the intensity of hemodynamic stress [17].

#### 2.4.4. Interplay with Small Vessel Disease and Neurovascular Uncoupling

The structural changes of cSVD, such as arteriolar wall thickening, perivascular space dilation, and capillary rarefaction [44,135], further impair the autoregulatory reserve [136,137]. Concurrent neurovascular uncoupling [138] reduces the precision of flow regulation in response to metabolic demand, exacerbating regional perfusion instability [139].

Moreover, orthostatic hypotension, nocturnal hypertension, and increased blood pressure variability, all common in older adults, can provoke repeated autoregulatory failure, progressively worsening vascular damage [140].

Importantly, autoregulatory failure is both a cause and a consequence of cSVD. The chronic ischemia, BBB leakage, and microvascular rarefaction characteristic of cSVD further erode vascular reactivity, creating a vicious cycle wherein structural damage and functional dysregulation exacerbate one another [15,137,141]. This interplay increases the likelihood of both progressive CMBs accumulation and catastrophic vessel rupture.

### 2.5. Chronic Sterile Inflammation—Inflammaging

Aging is associated with a state of chronic, low-grade systemic inflammation, termed “inflammaging” [142,143,144,145,146,147], which impacts the cerebral circulation as well. In aging endothelial cells acquire a pro-inflammatory phenotype [148,149,150]. The increased presence of senescent cells in the aging brain through the release of SASP contributes to a heightened state of inflammation [77,81,82,151]. Activated microglia and astrocytes release pro-inflammatory cytokines, further disrupting the BBB and promoting vascular injury. Elevated levels of circulating inflammatory mediators such as IL-6, TNF-α, and CRP have also been linked to increased burden of CMBs and higher risk of ICH in older adults.

The role of inflammation in hemorrhage formation is particularly relevant in CAA and has recently gathered increasing interest [37]. Indeed, CAA has been linked to chronic cellular and molecular inflammatory alterations in the brain both in experimental animals and in humans [152,153]. In addition, CAA can manifest in a typically subacute (but occasionally chronic) progressive encephalopathy termed CAA-related inflammation (CAA-RI), a condition histopathologically characterized by the accumulation of perivascular and/or transmural (i.e., vasculitic) inflammatory infiltrates in association with CAA-affected vessels, comprising lymphocytes, microglia/macrophages, and frequently multinucleated giant cells. The typical radiological presentation (in addition to CAA-compatible lobar hemorrhagic features) includes the appearance of asymmetric confluent white matter hyperintensities due to vasogenic edema. However, the most recent diagnostic criteria recognize the role of additional radiological features reflecting inflammation, including sulcal non-nulling on the fluid-attenuated inversion recovery (FLAIR) MRI and leptomeningeal contrast enhancement, representing a change in cerebrospinal fluid consistency and an increased leptomeningeal blood vessel permeability, respectively [154,155]. Importantly, remission of inflammation with clinical improvement can be achieved by immunosuppressive therapy (with corticosteroids in the pipeline) in ~80% percent of the cases; however, spontaneous improvement is also frequently reported (in some ~30–60% of the non-treated) [154]. Though early studies comparing CAA-RI and non-inflammatory CAA cohorts did not suggest an association between inflammation and ICH formation, a recent retrospective study on patients with sufficient follow-up reported strongly suggestive findings [154]. These include (1) a remarkable 33.7% incidence of lobar ICH at presentation or within 1 year in definite CAA-RI patients compared to the known ~8.5% annual lobar ICH risk in CAA in general [156], (2) a frequent direct topographic association of hallmark white matter changes with CMBs, a feature also being associated with increased probability of true vasculitic pathology, (3) and the observation that clinical improvement (suggesting remission of inflammation) was associated with a significantly decreased probability of lobar ICH development within 1 year [154]. Recent reports on devastating fatal ICH following systemic thrombolysis with post-mortem findings of silent CAA-RI are in line with this concept [157,158]. Most recently, a series of CAA patients presenting with “amyloid spells”, pathognomonic cSAH/disseminated CSS, and additional leptomeningeal enhancement (i.e., meeting the novel criteria for *probable CAA-RI*) demonstrated parallel improvement of the spells and the leptomeningeal enhancement, suggesting a possible link between cSAH/cSS formation and chronic inflammation in CAA [159]. With the border between inflammatory and non-inflammatory CAA becoming gradually diminished, clinical trials aiming at halting inflammation with the purpose of preventing lobar ICH development in CAA are underway [152].

**Figure 2:**
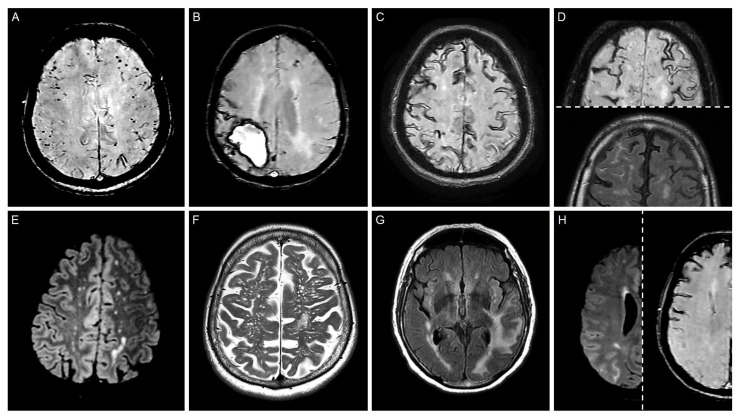
Hallmark magnetic resonance imaging features of CAA and CAA-RI. (**A**) Strictly lobar CMBs presenting as small globular hypointensities on SWI (a left parietal CSS can also be appreciated). (**B**) Lobar ICH (late subacute) in the right parietal lobe on SWI (several surrounding CSS, a small left parietal cSS, and two left frontal CMBs can also be observed). (**C**) Disseminated CSS in form of gyral hypointensities on the cortical surface on SWI in a patient presenting with TFNEs (a single CMB can also be appreciated right postcentrally). (**D**) Multiple foci of cSAH on the right prefrontal convexity presenting as a constellation of SWI hypointensity filling the affected sulci with corresponding SNN (i.e., sulcal hyperintensity) on the FLAIR (multiple chronic cSS can also be appreciated with no corresponding SNN). (**E**) White matter hyperintensities in a multispot pattern on FLAIR in the bilateral subcortical white matter, a feature introduced in the Boston criteria v2.0 [106]. (**F**) Severe MRI-visible (i.e., dilated) perivascular spaces in the centrum semiovale on T2, a feature introduced in the Boston criteria v2.0 [106]. (**G**) Asymmetric confluent white matter hyperintensity corresponding to vasogenic edema (also known as. ARIA-edema) in the left temporal, parietal, and occipital lobes on FLAIR in a patient with strictly lobar hemorrhagic alterations and subacute cognitive decline, meeting the classical criteria for probable CAA-RI (foci of thin SNN can also be appreciated; the edema completely resolved after corticosteroid therapy). (**H**) Right parietal SNN without corresponding SWI hypointensity (corresponding to ARIA-effusion) in a patient presenting with TFNEs and disseminated cSS, meeting the proposed extended criteria for probable CAA-RI [154]. (ARIA, amyloid-related imaging abnormality; CAA, cerebral amyloid angiopathy; CAA-RI, CAA-related inflammation; CMB, cerebral microbleed; cSAH, convexity subarachnoid hemorrhage; cSS, cortical superficial siderosis; FLAIR, fluid-attenuated inversion recovery; ICH, intracerebral hemorrhage; MRI, magnetic resonance imaging; SNN, sulcal non-nulling; SWI, susceptibility-weighted imaging; TFNE, transient focal neurological episode).

With the advent of monoclonal anti-β-amyloid antibodies as proposed and recently approved therapeutic options in AD, a group of radiological alterations has emerged in the past decade as adverse events in clinical trials, collectively termed amyloid-related imaging abnormalities (ARIAs) [160]. These comprise (1) ARIA-H (ARIA-hemorrhage), including lobar/subarachnoid hemorrhagic alterations typical of CAA, and (2) ARIA-E (ARIA-edema and ARIA-effusion), including white matter vasogenic edema and sulcal hyperintensity (i.e., sulcal non-nulling) on the FLAIR, respectively. Due to the striking resemblance of these alterations and their clinical symptoms to that seen in CAA-RI (especially as per the most recent criteria proposals [154], the identical (peri)vasculitic change of CAA vessels reported as histopathological correlate in the few autopsy reports available [161,162], the propensity to respond to steroid therapy, and the excess risk posed by carrying an *ApoE* ε4 allele, this adverse reaction has been referred to as iatrogenic CAA-RI or iatrogenic ARIA, as opposed to the spontaneous CAA-RI or spontaneous ARIA [163]. Iatrogenic ARIA has occasionally been associated with macroscopic lobar ICH in clinical trials, representing one of the most feared complications, sometimes in the context of systemic thrombolysis applied for presumed ischemic stroke [162,164]. Though the rigorously defined eligibility criteria (e.g., absence of prior spontaneous ARIA-H or ARIA-E and for lecanemab the absence of two *ApoE* ε4 alleles) will likely reduce the risk of such complications, heightened surveillance for ARIA and related clinical signs will be warranted in the upcoming monoclonal anti-β-amyloid antibody era to address this newly emerged etiology of lobar ICH. Figure 2 shows the hallmark magnetic resonance imaging features of CAA and CAA-RI.

### 2.6. Systemic Endocrine Dysregulation: Role of IGF-1 Deficiency

The vulnerability of the aging brain to hemorrhagic injury is not solely the result of local vascular changes. Increasing evidence suggests that aging is orchestrated, at least in part, by systemic regulatory mechanisms [43,44,45,46,165,166,167], circulating factors that influence the function and resilience of distant organs, including the brain [167]. This concept has been elegantly demonstrated in parabiosis studies, in which the circulatory systems of young and old animals are surgically joined [167]. Exposure of aged animals to young blood reverses multiple age-related impairments, including declines in cognitive function, neurogenesis, and vascular function, while young animals exposed to old blood exhibit premature vascular aging and reduced endothelial integrity [43,44,45,46,167,168].

These experiments have shifted the paradigm of aging research by revealing that circulating pro-youth or pro-aging factors can directly regulate cerebral vascular health [167]. Among these, IGF-1 has emerged as a central player in the systemic control of vascular aging [32,33,135,138,169,170,171,172].

IGF-1 is a pleiotropic growth factor predominantly produced in the liver under the control of growth hormone (GH), and its circulating levels decline markedly with age in both humans and experimental animals [138,172,173,174,175,176,177,178]. IGF-1 plays a crucial role in maintaining vascular homeostasis through multiple, interrelated mechanisms [172,179,180,181]. It supports endothelial function by enhancing the activity of endothelial nitric oxide synthase (eNOS), thereby promoting the production of NO, a key mediator of vasodilation and vascular tone [172,179,180,181,182]. In parallel, IGF-1 exerts antioxidant effects by modulating the generation of reactive oxygen species (ROS), improving mitochondrial function, and limiting oxidative stress within the vascular wall [38,172,177,179,180,181]. It also plays a central role in preserving BBB integrity, helping to stabilize endothelial tight junctions and suppress inflammatory signaling pathways that compromise barrier permeability [183,184]. Finally, IGF-1 facilitates vascular repair by promoting endothelial cell survival, stimulating proliferation, and enhancing angiogenesis [135,185,186,187,188,189,190]. Through these combined actions, IGF-1 maintains the structural and functional integrity of the cerebral microvasculature and protects against aging-related microvascular damage [32].

With aging, the decline in systemic IGF-1 [172] signaling leads to endothelial dysfunction, loss of BBB integrity [183], and increased microvascular fragility [191]—key contributors to both CMBs and ICH [138,183]. Experimental studies in IGF-1-deficient or IGF-1-depleted animal models consistently show an increased burden of CMBs [32,33,38], impaired autoregulatory function [121,192] and neurovascular uncoupling [193], pathological structural adaptation to hypertension [186,194] and heightened vulnerability to hemorrhagic injury [38]. Conversely, restoration of IGF-1 levels has been shown to ameliorate vascular dysfunction in aged mice. Human studies further support these findings: lower serum IGF-1 levels in older adults have been associated with increased microvascular pathologies, neurovascular uncoupling [138], poorer cognitive outcomes, and elevated risk of spontaneous ICH [195]. These associations persist even after adjusting for classical vascular risk factors, highlighting IGF-1 as a potential circulating biomarker and therapeutic target in hemorrhagic small vessel disease [172]. The connection between IGF-1 deficiency and CMBs/ICH formation underscores the importance of endocrine-vascular crosstalk in the pathogenesis of cerebrovascular aging [32,33,194]. Unlike structural vascular changes, systemic hormonal imbalances may be more amenable to therapeutic modulation, offering a promising avenue for preventive interventions in at-risk older populations.

## 3. Clinical Features and Challenges in Older Adults

ICH in older adults presents distinct clinical challenges stemming from atypical symptomatology, age-related anatomical patterns, diagnostic challenges, and poorer prognoses [196]. Diagnostic pitfalls arise from atypical clinical presentation and overlapping baseline conditions, often delaying critical imaging and treatment [197]. Key predictors of outcome in this population include hematoma characteristics, initial neurological status, and age-specific factors such as frailty and pre-existing conditions.

### 3.1. Atypical Presentations in Older Adults

ICH in older adults frequently presents with more severe symptoms and poorer prognosis compared to younger patients [198]. Older adults with ICH manifest non-specific symptoms such as acute confusional states, delirium, or unexplained falls, which can also be attributed to baseline cognitive impairment, multiple chronic illnesses, systemic infections, metabolic disturbances, or adverse drug effects [199]. Additionally, age-associated physiological alterations, such as modified neuroinflammatory mechanisms and compromised cerebral autoregulation, can mask typical clinical signs of ICH and complicate timely recognition and diagnosis, often resulting in worse outcomes [10].

### 3.2. Hemorrhage Location and Aging Patterns

The anatomical distribution of ICH in older adults reflects the influence of different underlying vascular pathologies [200]. As previously discussed, deep hemorrhages, involving the basal ganglia, thalamus, and brainstem, are strongly associated with hypertensive arteriopathy, as prolonged exposure to elevated blood pressure leads to degenerative changes in the small perforating arteries supplying these regions [201]. Deep hemorrhages often result in profound motor or sensory deficits due to the involvement of critical subcortical structures, and their clinical course may be further complicated by conditions such as hydrocephalus, particularly in the case of intraventricular hemorrhage [202].

On the other hand, lobar hemorrhages affecting the cerebral cortex and immediately subcortical white matter are closely associated with CAA [200], a degenerative vasculopathy characterized by the deposition of β-amyloid protein predominantly within the walls of cortical and leptomeningeal blood vessels, leading to vessel fragility and increased susceptibility to hemorrhage [203,204]. Importantly, CAA-related lobar hemorrhages tend to be recurrent and are often accompanied by cSS [106]. The pathogenesis of cSS include chronic hemosiderin (iron) deposition due to (likely repeated) episodes of cSAHs, arising from CAA-affected fragile superficial cortical/leptomeningeal small vessels [205]. The clinical relevance of cSAH/cSS is provided by their propensity to be the anatomical substrate of transient focal neurological episodes (TFNEs, also known as ‘amyloid spells’) and their association with a further increased risk of lobar ICH [157,206,207]. Moreover, lobar ICH is associated with a higher risk of acute symptomatic seizures [208]. There is also a well-established link between CAA and cognitive decline, highlighting the broader clinical implications of this vascular pathology in the aging brain [209].

### 3.3. Diagnostic Pitfalls

Diagnosis in older adults with ICH is frequently challenged by overlapping comorbidities, pre-existing neurocognitive dysfunction and a higher degree of frailty [210]. One significant diagnostic pitfall lies in the difficulty of distinguishing new neurological deficits from existing ones, especially for those already diagnosed with conditions like ischemic stroke, dementia, Parkinson’s disease, or chronic subdural hematoma [211]. Furthermore, cerebral atrophy in advanced age can reduce mass effect and mitigate intracranial pressure elevations, thereby attenuating clinical signs despite significant hemorrhage [212]. As a result, clinicians may not consider or underestimate the severity of ICH and delay crucial neuroimaging. Likewise, a relatively normal neurological assessment score, such as the National Institutes of Health Stroke Scale (NIHSS) or Glasgow Coma Scale (GCS), may not accurately reflect the underlying severity of injury in this age group [213]. A low threshold for the early use of neuroimaging, particularly non-contrast CT scans, is critical for distinguishing ICH from other possible causes in this age group.

### 3.4. Prognosis in Aging Populations

Advanced age is consistently associated with worse outcomes following ICH [214]. In addition to increased acute-phase mortality, older adults exhibit decreased functional recovery and are more likely to experience persistent disability and post-ICH cognitive decline [215]. The risk of fatal complications, such as recurrent ICH, expansion of the hematoma, aspiration pneumonia, or multisystem failure, is magnified by age-related physiological vulnerabilities and multiple comorbidities [216]. The cumulative burden of cSVD, including silent cerebral infarctions, white matter lesions, and CMBs, further compromises plasticity and post-stroke recovery [34]. Moreover, older adults have a higher likelihood of requiring long-term institutional care or assistance with basic activities of daily living [214].

### 3.5. Predictors of Outcome

Prognosis in older ICH patients is determined by a combination of clinical [217], genetic [218], radiological, and age-specific factors [219,220]. Established predictors of a worse outcome include larger hematoma volume [221], deep hematoma location [222], intraventricular extension, initial low level of consciousness [200], advanced age, and an anticoagulated state [105]. Regarding older adults, additional factors such as baseline frailty, cognitive impairment, and the degree of functional independence prior to ICH play a significant role [216]. Frailty, as assessed by validated tools like the Clinical Frailty Scale, independently predicts poor neurological recovery, longer hospitalizations, and a greater likelihood of discharge to long-term care [223]. Similarly, the presence of multiple CMBs and cSS may signal underlying CAA and portend a higher risk of recurrence and long-term cognitive impairment [106]. Comprehensive assessment is especially important in older ICH patients to guide acute management and rehabilitation [219].

## 4. Management Considerations

The management of ICH in older adults requires a comprehensive approach that integrates acute phase management, personalized treatment planning, and secondary prevention.

### 4.1. Acute Phase Care

Structured ICH care bundles have been introduced to support the timely and standardized delivery of acute-phase care [224]. These bundles include rapid triage and neuroimaging, intensive blood pressure control [225], immediate reversal of anticoagulation, neurosurgical consultation, and transfer to a dedicated stroke unit [226]. Care bundles such as the ABC-ICH protocol (Rapid Anticoagulant reversal, Intensive Blood pressure lowering, and a Care pathway for prompt neurosurgical referral) have demonstrated improved outcomes by reducing functional disability and in-hospital mortality, when embedded into daily practice [227]. The initial emergency response begins with immediate attention to the patient’s airway, breathing, circulation, and rapid neuroimaging [224]. Once the diagnosis of ICH is confirmed, care bundle protocols are implemented (“code ICH” as an analogy to “code stroke”) [228].

Antihypertensive treatment should be initiated in patients presenting within six hours of symptom onset with SBP ≥150 mmHg [229,230]. Although the benefit is less certain, treatment may still be considered in cases where symptom onset is ≥6 h or unknown if SBP is ≥150 mmHg [17,231]. The treatment target is an SBP ≤140 mmHg, which should be maintained consistently for seven days [229]. The first dose of antihypertensive medication should be administered within 30 min of hospital arrival, and the target SBP should be achieved within 60 min [17,231]. However, abrupt large SBP reductions of 60 mmHg within one hour should be avoided [232]. Notably, older adults, who may have impaired cerebral autoregulation, are at greater risk for hypoperfusion with aggressive SBP lowering and require more individualized approaches [233].

Immediate discontinuation and prompt reversal of anticoagulation are critical steps in the acute management of ICH, as continued anticoagulant activity may contribute to hematoma expansion and worse clinical outcomes [234]. Reversal protocols must be tailored to the specific type of anticoagulant involved, with a door-to-needle time of ≤30 min [231,235]. For patients on vitamin K antagonists with an international normalized ratio (INR) ≥1.3, reversal should include intravenous vitamin K and rapid administration of prothrombin complex concentrate (PCC) [236]. In contrast, patients receiving direct oral anticoagulants (DOACs) require agent-specific reversal strategies, such as idarucizumab for dabigatran [237], and andexanet alfa for factor Xa inhibitors like apixaban, rivaroxaban, or edoxaban [238,239]. PCC may be used as an alternative if a specific DOAC reversal agent is either unavailable or unlicensed [235]. Heparins can be reversed by protamine-sulphate [240]. Recombinant activated factor VII (rFVIIa) [241] and tranexamic acid may be considered as an adjunctive hemostatic agent in cases where specific reversal therapies are unavailable or contraindicated [242,243].

Surgical management of ICH in older adults, such as craniotomy or minimally invasive evacuation, should be made on a case-by-case basis by a multidisciplinary team within six hours [231]. The potential benefits of surgical intervention in this population must be carefully balanced against procedural risks, the likelihood of meaningful neurological recovery, the burden of postoperative rehabilitation, and the patient’s values and goals of care [244]. The decision to pursue surgical treatment must go beyond radiological findings and consider key age-related factors such as baseline functional status, cognitive reserve, comorbid conditions, and overall frailty [245]. Suggested criteria include patients with a pre-morbid modified Rankin Scale (mRS) score of ≤2 and a reasonable prognosis, along with one or more of the following: a GCS score ≤13, a supratentorial ICH volume ≥20 mL, posterior fossa ICH, or obstruction of the third or fourth ventricles [231,235]. The most likely candidates to benefit from minimally invasive surgery are both sexes, age of 30 to 80 years with superficial hematoma, GCS score of ≥9, hematoma volume between 25 and 40 mL, and undergoing surgery within 72 h of the symptom onset [246]. When the decision of hematoma evacuation has been made, the potential benefit of obtaining histological specimen of the leptomeninges and cortex to provide definite etiological diagnosis of a lobar ICH (with particular focus on CAA) should be kept in mind by neurosurgeons.

**Figure 3:**
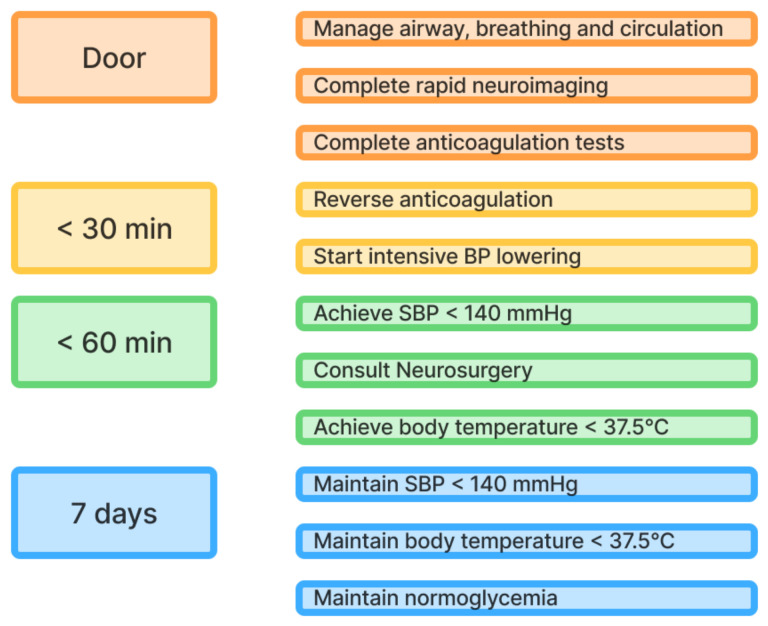
Acute care bundles for patients with intracerebral hemorrhage (BP, blood pressure; SBP, systolic BP).

ICH care bundle also includes maintenance of normothermia and normoglycemia together with normotonia up to 7 days [229,235,247]. Finally, consistent evidence supports the management of ICH patients in dedicated stroke units, which significantly improves outcomes by reducing mortality and long-term functional dependence compared to general medical wards [248]. The structured acute care bundles for intracerebral hemorrhage are summarized in Figure 3.

### 4.2. Tailored Strategies for Older Adults

Managing ICH in older adults requires a patient-centered approach that takes into account the complex interplay of age-related factors, biological frailty, pre-existing neurological and cognitive status, comorbidities, and, most critically, the patient’s goals of care [215]. The potential benefits and risks must be carefully considered when identifying patients who are most likely to derive meaningful benefit from more aggressive treatments [244]. Multidisciplinary evaluations involving geriatrics, neurology, neurosurgery and ethics teams are essential to determine appropriate treatment plans [249]. Beyond acute medical management, focused approaches for early mobilization, optimizing nutrition, preventing and managing delirium, and avoiding hospital-associated complications play a critical role in improving quality of life [231,235,250].

### 4.3. Secondary Prevention

**Figure 4:**
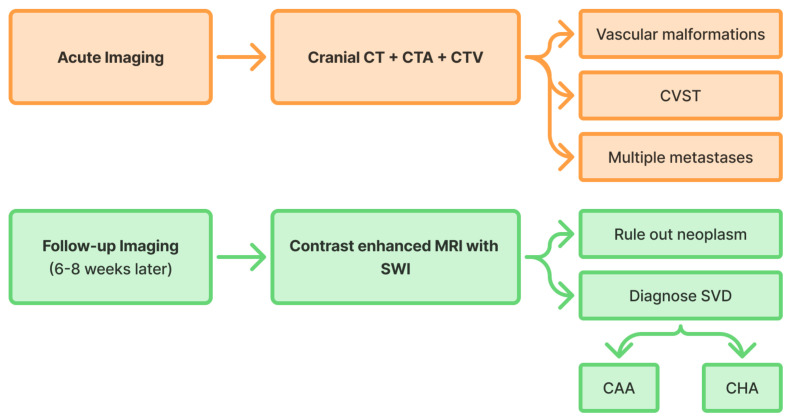
Proposed diagnostic work-up for lobar intracerebral hemorrhage. (CAA, cerebral amyloid angiopathy; CHA, cerebral hypertensive arteriopathy; CT, computed tomography; CTA, CT angiography; CTV, CT venography; CVST, cerebral venous sinus thrombosis; SVD, small vessel disease; SWI, susceptibility-weighted imaging).

The cornerstone of secondary prevention after ICH is adequate etiological diagnosis. Since the vast majority of deep ICH are likely to be secondary to hypertensive arteriopathy, the comprehensive diagnostic work-up is especially relevant for ICH with lobar location. After ruling out secondary (i.e., structural) etiologies in the acute setting (including vascular malformations and cerebral venous sinus thrombosis), a contrast enhanced MRI is to be performed to evaluate a possible underlying neoplasm. The optimal timing of MRI is uncertain, but ideally after a few (up to 6–8) weeks to allow for adequate visualization by regression and aging of the hematoma, with the most recent guidelines suggesting its repeat after three to six months if negative [235]. Once secondary etiologies are excluded, thus a primary, i.e., cSVD etiology is suspected, these MRI scans enable the etiological differential diagnosis of CAA and hypertensive arteriopathy, following the probable CAA diagnosis as per the v2.0 Boston criteria [106]. Figure 4 illustrates the proposed diagnostic work-up for lobar ICH, including acute-phase and follow-up imaging. Though most cases with mixed (i.e., lobar and deep) hemorrhagic alteration patterns are likely to be due to hypertensive arteriopathy only, addressing concomitant CAA in these patients with cerebrospinal fluid and plasma biomarkers as well as with positron emission tomography (PET) is of extensive research interest.

In detail, cerebrospinal fluid levels of β-amyloid_1–42_, β-amyloid_1–40_, and β-amyloid_1–38_ have been consistently found decreased compared to controls, reflecting cerebral β-amyloid deposition, with prominent vascular involvement. Since β-amyloid_1–40_ and β-amyloid_1–38_ are abundant in CAA vessels but less so in senile plaques, these decreases in β-amyloid_1–40_ and β-amyloid_1–38_ appear to be specific to CAA, and not characteristic to AD in general. On the other hand, the concentrations of Tau (as a general marker of neuronal loss) and phosphorylated Tau_181_ were generally higher compared to controls but lower compared to AD at the cohort levels, reflecting a subpopulation of CAA with concomitant advanced AD neuropathologic change [251,252]. Study on the plasma levels of β-amyloids and other core AD biomarkers have so far provided incoherent results [253,254]. The assessment of cerebral β-amyloid and aggregated Tau pathologies by PET can be reasonable alternative to cerebrospinal fluid analyses; however, evidence regarding aggregated Tau PET (by using the tracer [^18^F]T807) has only recently emerged [255,256]. Though the classical and clinically approved tracers of β-amyloid (such as [^11^C]PIB, [^18^F]florbetapir, [^18^F]florbetaben, and [^18^F]flutemetamol) cannot make distinction between vascular and parenchymal plaque pathology, a recently reported ligand [^18^F]K10-008 might provide a novel vascular-specific alternative in the future [257].

Hypertension remains the most significant modifiable risk factor for ICH recurrence, and its management is particularly critical in the older adults [34]. Regular monitoring and dose adjustments are essential to avoid complications such as orthostatic hypotension and cerebral hypoperfusion, which can lead to falls, syncope, or cognitive decline [258].

The resumption of antithrombotic therapy presents a complex clinical challenge and the risk of recurrent ICH versus thromboembolic events need to be weighed [259]. Factors that increase hemorrhagic risk are older age, already recurrent ICH, lobar ICH, higher number and lobar location of CMBs, CAA (especially when with cSS), uncontrolled hypertension and *ApoE* ε2 allele [260,261]. Restarting antiplatelet therapy for an ischemic vascular comorbidity seems safe in ICH survivors and not only decreases the risk of thromboembolic events but also that of recurrent hemorrhage [262,263]. Patients with an extremely high thromboembolic risk such as mechanical heart valves and left ventricular assistance devices require anticoagulant resumption as soon as possible, no later than two weeks [264]. Restaring anticoagulation in patients with atrial fibrillation requires more consideration [236]. For patients at a low to moderate risk of recurrent hemorrhage it is reasonable to resume oral anticoagulation after two months, however for those at a very high hemorrhagic risk left atrial appendage closure may be explored as an alternative [265]. Healthy diet, smoking cessation, alcohol moderation, and increased physical activity can also optimize vascular health [266]. In older adults with CAA or extensive lobar hemorrhagic alterations identified on SWI MRI, the risk of recurrent ICH may outweigh the benefits of anticoagulation [267]. These imaging biomarkers have become critical tools for stratifying hemorrhagic risk, yet there remains no consensus on precise thresholds for safe resumption of anticoagulants, highlighting the need for individualized decision-making [219].

### 4.4. Emerging Therapeutic Directions

Recent advances in understanding the vascular vulnerabilities associated with aging have opened promising paths for preventing and treating ICH and related CMBs. These emerging therapies target the biological mechanisms underlying vascular fragility and secondary brain injury, aiming to modify the disease course beyond traditional acute interventions [268].

Chronic neuroinflammation plays a crucial role in secondary injury following ICH [269]. Prolonged activation of microglia and astrocytes leads to the release of pro-inflammatory cytokines and oxidative stress, exacerbating edema, neuronal injury, and blood–brain barrier disruption [269,270]. Novel agents targeting this cascade, such as inhibitors of the NLRP3 inflammasome are under active investigation [271]. These therapies may reduce perihematomal damage and improve neurological recovery by dampening maladaptive inflammatory responses, particularly in older patients with heightened inflammatory vulnerability [272].

Senolytic therapies represent another innovative strategy, selectively targeting senescent glial and endothelial cells that accumulate with age and contribute to endothelial dysfunction and vascular instability [273]. Preclinical studies demonstrate that senolytics can restore vascular integrity, reduce blood–brain barrier disruption, and diminish hemorrhagic risk, providing a novel mechanism for neurovascular protection in aging brains [35,93].

Additionally, declining systemic levels of IGF-1 with aging have been implicated in weakening neurovascular structures [138]. Strategies to restore or mimic IGF-1 signaling may strengthen fragile cerebral vessels, enhancing resilience against hemorrhage [32]. This reflects a broader concept of targeting the biological aging process of the vasculature to prevent ICH [169].

In CAA, decreasing neuroinflammation by minocycline in part through decreasing MMP-2 and MMP-9 activity is currently of extensive research interest [152,274]. In addition, a multicenter phase 2 clinical trial with Mivelsiran, an intrathecally administered RNA interference therapeutic targeting the *APP* with the aim to decrease the load of pathological β-amyloid and thus the development of future lobar hemorrhagic alterations is underway (NCT06393712). While these approaches remain largely in experimental or early clinical stages, they signify an important shift toward preventative therapies focused on vascular health and biological aging regulation, rather than solely on acute hemorrhage management.

### 4.5. Ethical and Palliative Care Aspects

The management of ICH in frail older adults, especially those with limited life expectancy or low likelihood of meaningful neurological recovery, demands ethically grounded decision-making [275]. Clinicians must navigate the delicate balance between the potential benefits of aggressive interventions and the associated physical, emotional, and functional burdens [276]. The integration of palliative care becomes crucial when life-prolonging therapies are unlikely to improve outcomes or are against the patient’s goals of care [275,276]. Early palliative involvement focuses on symptom relief, psychosocial support, and ensuring patient comfort and dignity throughout the disease course [277]. Engaging in early discussions about advanced directives and goals of care allows patients and families to make informed decisions that align with their values and preferences [278].

## 5. Conclusions

ICH in the aging population arises from a complex interplay of vascular aging processes, including endothelial dysfunction, BBB breakdown, impaired autoregulation, chronic inflammation, and systemic endocrine dysregulation. These mechanisms collectively weaken the structural and functional integrity of the cerebral microvasculature, promoting both covert hemorrhage and subclinical lesions such as CMBs. Increasing evidence suggests that CMBs and ICH are not isolated phenomena but rather represent different manifestations along a shared continuum of hemorrhagic small vessel disease.

Despite the rising incidence of ICH, CMBs, and cSAH/cSS in older adults, this population remains underrepresented in clinical trials, limiting the applicability of current evidence to those most at risk. There is an urgent need to develop and validate biomarkers of vascular aging and rupture risk, which could support early detection, risk stratification, and individualized prevention strategies.

Future research should prioritize the mechanistic targeting of aging pathways, including interventions that restore endothelial resilience, stabilize cerebral autoregulation, correct systemic hormonal imbalances such as IGF-1 deficiency, and act against inflammatory alterations accompanied by cSVDs. Furthermore, growing recognition of the link between CMBs, ICH, and VCID highlights the importance of addressing long-term cognitive outcomes in ICH survivors.

Ultimately, advancing our understanding of how aging predisposes the brain to hemorrhagic injury will be essential to guide the development of age-aware, personalized therapeutic approaches. Integrating geroscience principles into stroke research may pave the way for more effective prevention, better post-ICH care, and improved outcomes for older adults at risk of cerebrovascular decline.

## Data Availability

Not applicable.
